# Risk factors for early mortality and impaired quality of life in oral cavity cancer – head and neck cancer register study

**DOI:** 10.2340/1651-226X.2025.43469

**Published:** 2025-07-03

**Authors:** Teija Nieminen, Morag Tolvi, Tuija Ylä-Kotola, Lasse Lehtonen, Antti Mäkitie, Taru Ilmarinen

**Affiliations:** a Department of Perioperative and Intensive Care Medicine, University of Helsinki and Helsinki University Hospital, Helsinki, Finland; b Research Program in Systems Oncology, Faculty of Medicine, University of Helsinki, Helsinki, Finland; c Department of Otorhinolaryngology – Head and Neck Surgery, University of Helsinki and Helsinki University Hospital, Helsinki, Finland; d Department of Plastic Surgery, University of Helsinki and Helsinki University Hospital, Helsinki, Finland; e HUS Diagnostic Center, Helsinki University Hospital and Department of Public Health, University of Helsinki, Helsinki, Finland

**Keywords:** Surgery, risk factors, mortality, curative-intent, health-related quality of life, EORTC QLQ-H&N35

## Abstract

**Background and purpose:**

Treatment of locoregionally advanced oral cavity cancer (OCC) is associated with treatment-related complications, functional deficits, and even early mortality. High-quality register data could help in choosing between curative and non-curative intent treatment options.

**Materials and methods:**

The Helsinki Head and Neck Cancer Register (HHNCR) is linked with the EORTC QLQ-H&N35 questionnaire automatically sent to all patients at diagnosis and predetermined intervals. We analyzed pretreatment data of all patients diagnosed with OCC during 2018–2023, focusing on risk factors for early mortality and impaired health-related quality of life after curative-intent treatment.

**Results:**

Of 597 patients, 556 (93%) were treated with curative intent. Thirty-nine (7.0%) patients died within 6 months after diagnosis. The independent risk-factors for 6-month mortality identified in multivariable analysis were T3 stage (OR 8.3 [2.6–26.5], *p* < 0.001), T4 stage (OR 8.2 [2.5–26.8], *p* < 0.001), N3 stage (OR 10.6 [3.2–35.1], *p* < 0.001), and Adult Comorbidity Evaluation (ACE)-27 score 2–3 (OR 5.5 [2.4–12.5], *p* < 0.001). These risk-factors were used to create a predictive risk score for early death. Younger, healthier patients had significantly higher EORTC QLQ-H&N35 response rates compared with older patients with comorbidities. Six months after diagnosis, patients with a stage III–IV tumor had significantly higher scores in 15 of 18 items, compared with patients with a stage I–II tumor.

**Interpretation:**

Early mortality was associated with advanced tumor (T) and nodal (N) stage, and increased pretreatment comorbidity (ACE-27) scores. The strongest predictor for impaired quality of life was locoregionally advanced disease.

## Introduction

Head and neck cancer (HNC) is the seventh most common cancer type in the world [1]. More than 800,000 HNCs are diagnosed worldwide every year. HNC represents a heterogenous group of malignancies. The primary treatment approach varies depending on the affected subsites, which further affects patient outcome. In HNC patient populations, 6-month mortality after treatment with curative intent is highest in hypopharyngeal cancer, and second highest in OCC [2].

Oral cavity cancer (OCC) is the 16th most common cancer in the world [3]. The standard treatment of OCC typically involves surgery, radiation therapy, chemotherapy, or a combination of these, depending on the stage and extent of the disease [4]. Due to treatment-related side effects and complications, the outcome is often associated with impaired functional status and quality of life. Previous studies have shown that patients with HNC and with increased age and poor functional status, have higher risk of early mortality that is less than 6 months after diagnosis [5–7]. To reduce unnecessary treatment-related morbidity, a limited set of relevant pretreatment parameters should be defined and assessed separately for different head and neck sites.

Cancer databases and registries provide access to healthcare data that can be used to study epidemiological trends and to improve treatment decisions [8]. However, there is no consensus regarding the pretreatment parameters that should be routinely reported in HNC, and the potential biases in reporting and analyzing register data. Access to high quality register data would enable web-based tools to be used for more individualized treatment decisions [9, 10].

Previous studies on early mortality after surgery, and postoperative oncological treatment in HNC are limited [11, 12]. Some studies have reported on early postoperative mortality in OCC, defined as 30 days after surgery. In these studies, the mortality rate was 1.0% [13, 14]. In the study by Guan et al., 3.6% of patients with oral tongue cancer died within 3 months of cancer diagnosis [15].

Compared with other HNC patients, those with OCC may experience reduced health-related quality of life (HRQOL) due to limited mouth opening, and problems related with swallowing, chewing, speech, and saliva secretion. A recent systematic review and meta-analysis by Yuwanati et al. found reduced quality of life in patients with OCC compared with healthy individuals [16]. Extensive surgery may cause unnecessary functional decline or even reduce life expectancy in patients with significant comorbidities, increased age, or locoregionally advanced tumors. However, if speech and swallowing are already significantly impaired at the time of diagnosis, surgery may not further diminish quality of life but rather improve the management of oral functions and relieve pain.

The quality of life of HNC patients has been studied using HNC-specific patient-reported outcome measures (PROMs). In a systematic review, Faria et al. conducted an analysis of 115 studies. The researchers found that content mapping of the more common PROM measures showed variability and was weak in capturing symptom coverage, despite validation appearing to be satisfactory [17]. This suggests that further research is needed to obtain an accurate and reliable picture of the patient’s symptoms and problems.

The aim of our study is to identify which patients with OCC may not benefit from curative-intent treatment, either because of very short life expectancy or increased treatment-related morbidity. We report patient- and tumor-related pretreatment parameters of the Helsinki Head and Neck Cancer Register (HHNCR) in order to analyze their association with early mortality, using patients with OCC as a pilot cohort. Risk factors for impaired HRQOL are assessed using a register-integrated questionnaire (as measured with EORTC QLQ-H&N35[18]) among OCC survivors.

## Materials and methods

### Pretreatment parameters of the Helsinki Head and Neck Cancer Register

In Finland, more than 950 HNCs are reported annually, of which about 400 occur in the oral cavity [19]. The catchment area for HNC at Helsinki University Hospital encompasses approximately 2.2 million, representing about 40% of Finland’s population. Treatment recommendations for all newly diagnosed HNCs are given by a weekly multidisciplinary team (MDT) meeting and are based on a national treatment protocol, which is updated annually by the National Head and Neck Oncology Working Group.

The HHNCR was initiated in May 2018 to record relevant data of all new HNC cases at Helsinki University Hospital. After each MDT meeting, pretreatment data for each newly diagnosed patient including age, sex, International Classification of Diseases 10^th^ revision (ICD-10) classification, tumor, nodes, and metastases (TNM) classification, tumor stage, date of diagnosis, Adult Comorbidity Evaluation-27 (ACE-27) score, WHO performance status (PS) score, data on smoking and alcohol use are reported using an electronic form. The Finnish Cancer Register receives data on all new cancer cases automatically from the HHNCR and also directly from all pathology laboratories in the country [19].

To record HNC-specific PROMs, the register has been linked with the EORTC QLQ-H&N35 questionnaire. Since 2020, the questionnaire has been automatically activated for each patient before the MDT meeting, and thereafter at 6, 12, and 24 months. At each time point, the patient receives a text message, asking to apply a link and, after strong electronic identification, to fill in the questionnaire electronically.

### Patient data

To assess the quality of the register data, we reviewed the electronic health records of all patients diagnosed with HNC cancer and discussed at the MDT meeting in 2019 (*n* = 375), which was the first full calendar year of the register. We compared the electronic health records of all patients with OCC (*n* = 160) to register data and confirmed that the accuracy for all the aforementioned pretreatment parameters was over 95%. Thus, no systematic errors occurred in the data reported either manually or through data interfaces.

To assess pretreatment parameters affecting early mortality, the next step involved data search on all OCC cases reported to the HHNCR between May 2018 and December 2023. Each patient was followed for a minimum of 6 months after diagnosis or until death. A minimum requirement for inclusion was availability of ID number, age, sex, ICD code for OCC, date of diagnosis, and TNM stage. Dates of death are automatically reported to the register through an interface with Statistics Finland. EORTC QLQ-H&N35 data, ACE-27 and WHO PS, smoking and alcohol history were included in the analyses when available.

To assess the development of data coverage, we compared the number of patients reported within two time periods: years 2018–2021 and 2022–2023. These numbers were compared to the average annual number of new OCCs reported to the Finnish Cancer Register from all pathology laboratories of the Helsinki University Hospital catchment area.

### Health-related quality of life (EORTC QLQ-H&N35)

The EORTC QLQ-H&N35 questionnaire comprises 35 questions. These indicate the extent to which the patient experienced symptoms or problems specific to HNC during the past week. Patient responses are transformed to seven multi-item scales and 11 single items. Thirteen of the items are transformed to a scale of 0–100. For example, a response to a single item question ‘Have you had problems opening your mouth wide?’ gives either 0 (‘Not at all’), 33 (‘A little’), 66 (‘Quite a bit’), or 100 (‘Very much’) points. Five items are based on a dichotomous patient response (YES or NO).

### Statistical analyses

Statistical analyses were performed using IBM SPSS 29 for Windows (IBM Corp., Armonk, NY). Continuous variables were compared using the Mann–Whitney *U* test, and categorical variables using the chi-square or Fisher’s exact test. Two-sided p-values were computed, and a difference was considered statistically significant at *p* < 0.05. Univariable and multivariable logistic regression analyses were used to investigate predictors of death at 6 months after diagnosis. Predictors of early death with *p* < 0.05 in univariable analysis were entered into multiple logistic regression. The Kaplan–Meier method with Log rank test served to compare 6-month survival between risk groups.

Institutional permission to conduct this study was granted by the Research Administration of the HUS District (HUS/419/2018 and HUS/124/2023). This research involved only patient chart data, and therefore, no formal Research Ethics Board approval or informed consent was needed according to Finnish legislation (Medical Research Act 488/1999) [20].

## Results

### Pretreatment parameters associated with early mortality

In total, 597 patients with OCC were registered for the period 2018–2023. Of 597 patients, 556 (93.1%) were treated with curative intent. Of the 556 patients treated with curative intent, 39 (7.0%) died within 6 months of diagnosis. In univariate analyses, early death in patients with curative-intent treatment was significantly associated with T stage (*p* < 0.001), N stage (*p* < 0.001), overall stage (*p* < 0.001), ACE-27 score (*p* < 0.001), and WHO PS score (*p* < 0.001) but not with age (as a continuous variable), sex, smoking, or alcohol (Table 1).

**Table 1:** Demographics of all patients with curative-intent treatment, and factors associated with 6-month survival.

Demographics	All patients (*n* = 556)	Died within 6 months (*n* = 39)	Alive at 6 months (*n* = 517)	*p* ^a^
Age, median (range)	68 (19–98)	71 (24–98)	67 (19–96)	0.211^a^
Sex				0.621
Male, *n* (%)	305 (54.9)	23 (59.0)	282 (54.5)	
Female, *n* (%)	251 (45.1)	16 (41.0)	235 (45.5)	
T stage *n* (%)				**< 0.001**
T1	251 (45.1)	5 (12.8)	246 (47.6)	
T2	141 (25.4)	5 (12.8)	136 (26.3)	
T3	86 (15.5)	16 (41.0)	70 (13.5)	
T4	78 (14.0)	13 (33.3)	65 (12.6)	
N stage *n* (%)				**< 0.001**
N0	427 (76.8)	18 (46.2)	409 (79.1)	
N1	48 (8.6)	4 (10.3)	44 (8.5)	
N2	52 (9.4)	7 (17.9)	45 (8.7)	
N3	29 (5.2)	10 (25.6)	19 (3.7)	
Stage *n* (%)				**< 0.001**
I	235 (42.3)	5 (12.8)	230 (44.5)	
II	108 (19.4)	3 (7.7)	105 (20.3)	
III	84 (15.1)	9 (23.1)	75 (14.5)	
IV	129 (23.2)	22 (56.4)	107 (20.7)	
ACE-27 score, *n* (%)				**< 0.001**
0	148 (26.6)	7 (17.9)	141 (27.3)	
1	221 (39.7)	6 (15.4)	215 (41.6)	
2	139 (25.0)	13 (33.3)	126 (24.4)	
3	48 (8.6)	13 (33.3)	35 (6.8)	
WHO PS score, *n* (%)				**< 0.001**
0	144 (25.9)	2 (5.1)	142 (53.4)	
1	94 (16.9)	7 (17.9)	87 (32.7)	
2	33 (5.9)	5 (12.8)	28 (10.5)	
3	11 (2.0)	2 (5.1)	9 (3.4)	
4	1 (0.2)	1 (2.6)	0 (0.0)	
Missing	273 (49.1)			
History of smoking, *n* (%)				0.737
Never	214 (38.5)	13 (33.3)	201 (42.1)	
Former	115 (20.7)	8 (20.5)	107 (22.4)	
Current	185 (33.3)	15 (38.5)	170 (35.6)	
Missing	42 (7.5)			
Alcohol misuse, *n* (%)				0.237
Never	258 (46.4)	17 (43.6)	241 (76.3)	
Current or former	84 (15.1)	9 (23.1)	75 (23.7)	
Missing	214 (38.5)			
Primary treatment				0.615
Surgery	541 (97.1)	37 (92.5)	504 (97.5)	
Radiotherapy	6 (1.1)	2 (5.0)	4 (0.8)	
Chemoradiotherapy	9 (1.6)	0	9 (1.7)	
Adjuvant treatment				0.287
None	382 (68.6)	30 (75.0)	352 (68.1)	
Postoperative radiotherapy	117 (21.0)	3 (7.5)	114 (22.1)	
Postoperative chemoradiotherapy	57 (10.2)	6 (15.0)	51 (9.9)	

ACE-27 score: Adult Comorbidity Evaluation; WHO PS: World Health Organization Performance Status; T: primary tumor; N: lymph node.

a Mann–Whitney *U*-test (Chi-square test in all others).

Significant values are given in bold.

Multivariate binary logistic regression analyses included all predictors from the univariate analyses with *p* < 0.05, and age distributed into four groups (percentiles). In the multivariable model, age ≤ 57 years, T stage > 2, N stage > 2, and ACE score 2–3 were confirmed as independent risk factors for early death. Thus, patients with T3 and T4 stage tumors were eight times more likely to die within 6 months (T3 stage OR 8.3 [2.6–26.5], *p* < 0.001, T4 stage OR 8.2 [2.5–26.8], *p* < 0.001). Patients with N3 stage had 10 times higher odds of dying within 6 months (OR 10.6 [3.2–35.1], *p* < 0.001), and in patients with ACE-27 score 2–3, the odds were over five times higher (OR 5.5 [2.4–12.5], *p* < 0.001) (Table 2).

**Table 2:** Factors associated with early death (within 6 months of diagnosis) by multivariable binary logistic regression analysis.

Factors	Adjusted OR (95% CI)*	Significance (*p*)
Age		
≤ 57	1	**0.025**
58–68	0.201 (0.056–0.721)	**0.014**
69–75	0.995 (0.313–3.167)	0.993
≥ 76	1.308 (0.397–4.313)	0.659
Sex	0.817 (0.368–1.811)	0.618
ACE-27 score		
0–1	1	
≥ 2	5.472 (2.403–12.459)	**< 0.001**
T stage		
T1	1	
T2	1.838 (0.499–6.776)	0.360
T3	8.308 (2.608–26.464)	**< 0.001**
T4	8.176 (2.492–26.825)	**< 0.001**
N stage		
N0	1	**0.002**
N1	1.196 (0.352–4.060)	0.774
N2	1.983 (0.654–6.014)	0.227
N3	10.608 (3.210–35.050)	**< 0.001**

* Adjusted for other variables included in the model.

ACE-27 score: Adult Comorbidity Evaluation; OR: odds ratio; T: primary tumor; N: lymph node; CI: confidence interval.

Significant values are given in bold.

Primary treatment, postoperative complications, and disease progression in patients who died within 6 months after diagnosis are presented in Table 3.

**Table 3:** Summary of surgical and medical complications and tumor recurrences in 39 patients with oral cavity cancer who died within 6 months.

After curative-intent treatment	Early mortality *n* = 39
Age, median (range)	71 (24–98)
Surgical complications, *n* (%)	
No, *n* (%)	21 (53.8)
Yes, *n* (%)	18 (46.2)
Surgical site infection	6 (15.4)
Flap failure (or revision)	4 (10.3)
Fistula	4 (10.3)
Surgical site hematoma	3 (7.7)
Nerve damage	2 (5.1)
Medical complications, *n* (%)	
No, *n* (%)	20 (51.3)
Yes, *n* (%)	19 (48.7)
Pneumonia	10 (25.6)
Delirium	7 (17.9)
Arrhythmia	5 (12.8)
Prolonged mechanical ventilation	5 (12.8)
Atelectasis/respiratory failure	2 (5.1)
Re-admission to ICU	2 (5.1)
Heart failure	2 (5.1)
No complications, *n* (%)	13 (33.3)
Primary surgery, *n* (%)	36 (92.3)
Primary radiotherapy, *n* (%)	3 (7.7)
Postoperative CRT, *n* (%)	5 (12.8)
Postoperative RT, *n* (%)	6 (15.4)
Tumor recurrence, *n* (%)	
No	27 (69.2)
Yes	12 (30.8)
Local/regional	4 (10.3)
Distant	8 (20.5)
Free flap reconstruction	27 (69.2)

ICU: intensive care unit; CRT: chemoradiotherapy; RT: radiotherapy.

To stratify risk for early mortality, a pretreatment predictive scoring was created based on risk factors identified in the multivariable logistic regression analysis. Thus, T2–3 stage, N3 stage, and ACE-27 score 2–3 added 1 point each. Accordingly, the minimum score for an individual patient is 0 points, and the maximum score is 3 points. Considering the number of received points, patients were stratified into four risk groups: none (0 points), low (1 point), intermediate (2 points), and high risk (3 points).

**Figure 1:**
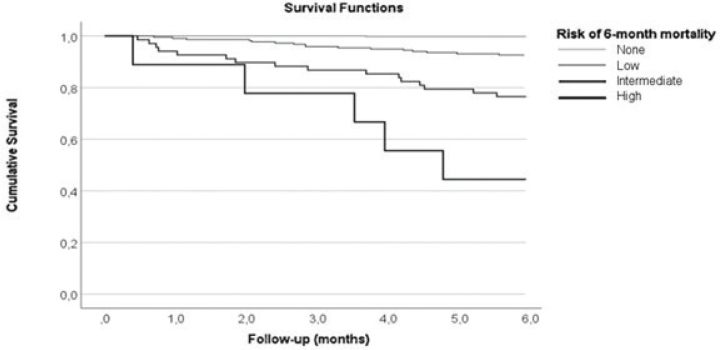
Six-month overall survival in 556 patients treated with curative intent. Based on the risk factors identified in the multivariable logistic regression analysis, a predictive risk score was calculated for each individual patient. Thus, T2–3 stage, N3 stage, and ACE-27 score 2–3 added 1 point each. Considering the number of received points, patients were stratified into four risk groups: none (0 points), low (1 point), intermediate (2 points), and high risk (3 points).

The distribution of overall survival time according to the pretreatment predictive score is presented in Figure 1.

### Data coverage

Of all the 597 study patients, 326 (55%) were diagnosed between years 2018 and 2021, and 271 (45%) between years 2022 and 2023. According to the Finnish Cancer Register, the average number of new OCC cases in the Helsinki University Hospital catchment area since 2018 is approximately 150 per year. Thus, the data coverage of the HHNCR had increased from 62.1% (2018–2021) to 90.3% (2022–2023). No statistically significant differences emerged between patients diagnosed during the early and the late period in terms of age, sex, T stage, N stage, M stage, overall stage, comorbidity score (ACE 0–1 vs. 2–3), PS score (WHO PS score 0–1 vs. 2–3), early death, treatment intent, or history of smoking or alcohol use.

### Quality of life

In total, 445 patients were asked to fill in the EORTC QLQ-H&N35 questionnaire by using an electronic form. Of the 445 patients, 235 patients (53%) responded either at diagnosis, 6 months after diagnosis, or at both time points. Younger age at diagnosis, lower comorbidity score (ACE-27 0–1), and better PS score (WHO PS score 0–1) were significantly associated with higher response rate both at diagnosis and at 6 months (*p* < 0.05). Patients with heavy alcohol consumption (previous or current) were less likely to respond. Sex, smoking history, and tumor stage were not associated with response rates. Only 14 of 49 patients (either curative or non-curative intent of treatment) who died within 6 months responded to the questionnaire at diagnosis. Thus, the response rate was too small to reliably analyze the association between pretreatment quality of life parameters and early death.

**Figure 2:**
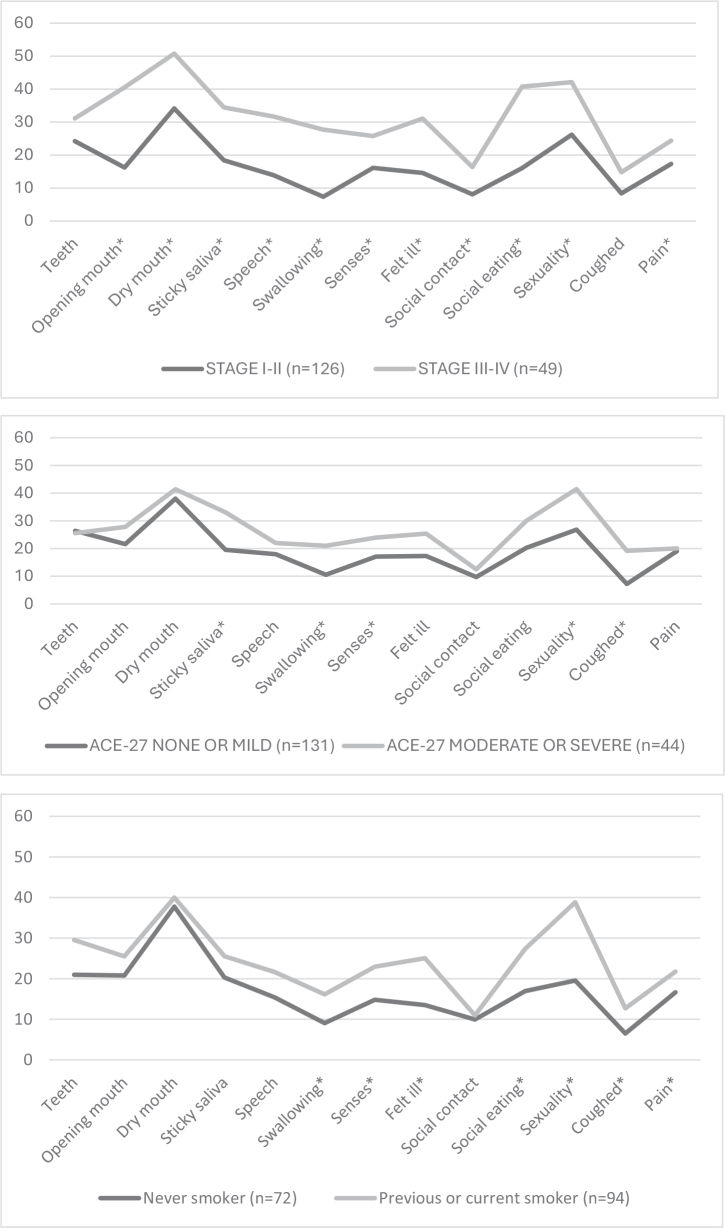
Mean scores of all EORTC QLQ-H&N35 items at 6 months reported on a scale from 0 to 100 by stage, ACE-27 score, and smoking history. Statistically significant differences between groups (Mann–Whitney *U* test, *p* < 0.05) are marked with an asterisk.

In EORTC QLQ-H&N35, patients treated for a stage III–IV tumor had significantly higher scores in 15 of 18 items at 6 months after diagnosis, compared with patients with a stage I–II tumor. Figure 2 presents mean scores of all items reported on a scale from 0 to 100 by stage, ACE-27 score, and smoking history.

Age and sex were not significantly associated with any of these items. Patients with WHO PS score 2–3 had significantly higher scores in items ‘Feeling ill’ (*p* = 0.009) and ‘Coughed’ (*p* = 0.003). Patients with heavy use of alcohol had significantly higher scores in ‘Swallowing’ (*p* = 0.042), ‘Senses’ (*p* = 0.042), ‘Feeling ill’ (*p* = 0.018), ‘Social eating’ (*p* < 0.001), ‘Coughed’ (*p* = 0.001), and ‘Pain’ (*p* = 0.042).

Dichotomous items (*n* = 5) are presented in Table 4 by stage, ACE-27 score, and smoking history. Sex, age, or heavy use of alcohol were not significantly associated with the dichotomous items. Patients with WHO PS score 2–3 had significantly more weight loss (*p* = 0.04), compared with patients with WHO PS score 0–1.

**Table 4:** Five items in EORTC QLQ-H&N35 questionnaire are based on a dichotomous patient response (YES or NO) at six months. Statistically significant differences between groups (Chi square, *p* < 0.05) are marked with an asterisk.

	Painkillers			Nutritional supplements			Feeding tube			Weight gain			Weight loss		
	Yes	No	*p*	Yes	No	*p*	Yes	No	*p*	Yes	No	*p*	Yes	No	*p*
Stage I–II	35	87	0.047*	21	102	0.025*	6	117	< 0.001*	20	101	0.98	24	97	< 0.001*
Stage III–IV	23	25		16	33		17	32		8	41		26	23	
ACE-27 0–1	47	79	0.23	28	100	0.84	15	113	0.28	20	107	0.66	37	90	0.90
ACE-27 > 1	11	33		9	35		8	36		8	35		13	30	
Never smoker	25	45	0.92	14	57	0.63	4	67	0.015*	10	60	0.36	13	58	0.005*
Previous or current	30	61		21	71		17	75		18	73		35	55	

EORTC QLQ-H&N35: European Organization for Research and Treatment of Cancer Quality of Life Questionnaire Head and Neck; ACE-27 score: Adult Comorbidity Evaluation.

## Discussion

In this study, the early mortality rate, that is within 6 months after diagnosis, for patients with OCC and curative-intent treatment, was 7.0%. We identified T3–4 stage, N3 stage, and ACE-27 comorbidity score 2–3 as independent risk factors for early death. We created a pretreatment predictive risk score to better illustrate the risk of early death. Over half of the patients with a risk score of 3 (T3–4 stage, N3 stage, and ACE-27 comorbidity score 2–3) died within 6 months. Pretreatment risk scoring may provide a useful tool for decision making in individual patients with an advanced stage tumor and significant comorbidities.

Previous studies on early mortality after OCC treatment are limited. In the studies by Talani et al., 6-month mortality rate in HNC was 4.5%, and 4.3% in OCC [2, 6]. In our study, the 6-month mortality rate was 7.0%, which may indicate that patients with poor prognosis were more often treated with curative intent.

In the study by Schwam et al., the only risk factor for 30-day mortality in OCC was steroid use for chronic conditions [13]. Significant independent predictors of 30-day mortality in OCC on multivariate analysis were age > 65, elevated comorbidity index according to the Charlson–Deyo comorbidity index, and stage T 2–4 disease [14]. A higher preoperative Charlson Comorbidity Score was associated with an increased risk of death 3 months postoperatively after curative intent treatment of oral cancer [21]. Oral and oropharyngeal cancer patients with ACE-27 score of 3 had lower survival rates [22]. In our study, older age at diagnosis was not associated with 6-month mortality.

In HNC, there is no consensus on optimal register content or key pretreatment parameters regarding comorbidities, patient performance, quality of life, nutritional status, or treatment delays. The structure of the healthcare system varies from country to country and hospital information systems are different. In the first years, our register underwent changes and updates, data content was refined, and staff were trained to use the register. Manual data collection is slow, especially for HNCs, which form a heterogeneous group of diseases. Over the past 3 years, the data coverage of our register has improved. The HHNCR coverage in 2022 was 93.2% when compared to the information from the Finnish Cancer Register. Our data coverage was improved by providing tutorials, by focusing on carefully selected, meaningful variables, and by reducing the need for manual work through the development of data interfaces. Also, clinicians have a statutory obligation to report all new cancer cases to the Finnish Cancer Register.

Creating high standard quality registers enables the development of value-based health care. The Swedish Head and Neck Cancer Register (SweHNCR) created in 2008, is an example of a well-established HNC quality register [23–25]. Similarly, the DAHANCA (Danish Head and Neck Cancer Group) register, established to collect comprehensive data on HNC patients in Denmark, is recognized as an excellent example of a high-quality clinical cancer register. It plays a crucial role in supporting clinical research and value-based health care through standardized, nationwide data collection [26, 27]. However, creating a high-quality head and neck oncology patient register that achieves comprehensive value-based health care is still challenging [28]. HHNCR was initiated, developed, and financed by Helsinki University Hospital, and currently covers approximately 40% of all HNC cases diagnosed in Finland. The register is not used in other Finnish hospitals or considered for nationwide use. National cancer register development in Finland is towards automatic data collection from electronic medical records in each public hospital through data interfaces with the Finnish Cancer Register. The goal is high data coverage without local software solutions and manual reporting of data. Our experience with the HHNCR can help to define which parameters should be prospectively collected across the country and used to measure different aspects of quality in HNC care.

Oncological outcomes and survival should not be used as sole indicators of quality. There is a lack of consensus on how clinical cancer registers should monitor post-treatment quality of life. In our register, data collection is automatic with web-based questionnaires sent to all patients at diagnosis and predetermined intervals after treatment. This allows inclusion of all patients at fixed time points regardless of where and when their clinical follow-up is organized. However, we found that older patients, and those with significant comorbidities, heavy alcohol use, and impaired functional status were less likely to respond. Furthermore, the response rate of patients who died within 6 months was very low at diagnosis. Due to the discrepancy in response rates, our EORTC QLQ-H&N35 results 6 months after diagnosis are not representative for all OCC patients. Lower response rates in older patients and those with significant comorbidities may explain why the differences in EORTC QLQ-H&N35 items were not statistically significant between age groups, and significant in only five out of the 18 items when comorbidity groups (ACE-27 0–1 vs. 2–3) were compared. To ensure higher response rates in older patients with comorbidities, it may be necessary to record the quality of life of selected patients during clinical follow-up visits. Clinicians should have insight into how even the frailest OCC patients are feeling and functioning.

To our knowledge, this is the first study assessing automatic, electronic, register-integrated QOL assessment in OCC. Further research is needed on how the quality of life at diagnosis should be measured in a register setting, and which parameters should be considered in treatment decisions. The EORTC QLQ-H&N35 questionnaire is quite extensive, and it may not be applicable for older, frail patients. More patient friendly and simple tools are needed for QOL assessment. Some previous studies have only utilized selected items of EORTC QLQ-H&N35 to more specifically assess treatment related morbidity, for example ability to restore oral diet, likelihood for permanent feeding tube, or issues with postoperative chronic pain [29, 30].

Some questions in EORTC QLQ-H&N35 are irrelevant for patients without teeth, without oral diets, or for patients who primarily have reduced oral communication skills due to other comorbidities. Discussing responses to the EORTC QLQ-H&N35 questionnaire, and different aspects of life quality during clinical follow-up visits may increase response rates and encourage patients to respond also later during follow-up.

We found that patients with a stage III–IV tumor had significantly higher scores in 15 of 18 items 6 months after diagnosis, compared with patients with a stage I–II tumor. Not surprisingly, the strongest predictors for QOL impairment were locoregionally advanced disease and large primary tumors. Of our surviving patients with T3–4 stage tumors 35% were feeding tube dependent, 48% needed pain killers, and 53% experienced weight loss at 6 months.

A recent systematic review and meta-analysis including 12 studies on HRQOL showed significantly impaired overall HRQOL in patients with OCC compared with the healthy population. Regarding the timing of QOL assessments, the review did not specify whether the evaluations were conducted before or after treatment. The authors concluded that more research is needed on HRQOL comparing pre- and postoperative treatment outcomes [16].

The present study has both strengths and limitations. Typically, it takes time to introduce a quality register for all healthcare professionals treating HNC. Our register was revised and improved in many aspects during the first years to be more user friendly, and the data coverage has steadily increased from 2018 to 2023. Despite the lower data coverage during the first years, we consider our study group to be a representative sample of all OCC patients treated at our hospital. Also, the present findings on pretreatment parameters associated with early death are in line with the previous studies. Using pretreatment comorbidity and PS scores in a quality register remains essential. In our register, data on WHO PS score and alcohol history were often missing. WHO PS score was therefore not included in the multivariable analysis. Data on heavy alcohol consumption was not available in over one-third of patients. Thus, the lack of a significant association with early mortality should be interpreted with caution.

## Conclusion

In summary, we concluded that early mortality after diagnosis of OCC and curative-intent treatment is influenced by higher tumor stage, comorbidity (ACE-27), and WHO PS scores. The strongest predictor for impaired quality of life was locoregionally advanced disease. Measuring HRQOL should be part of the evaluation of OCC treatment. Pretreatment risk scoring may provide a useful tool for decision making in individual patients with an advanced-stage tumor and significant comorbidities.
